# Near-full-length genome sequence of HIV-1 strain BF14, a G/K/J unique recombinant form, from an ART-suppressed patient in Ghana

**DOI:** 10.1128/mra.00913-26

**Published:** 2026-10-14

**Authors:** Solomon K. Amuzu, Heidi Meeds, Pious Appiah, Isaac K. Sraku, Ibrahim Jamfaru, Lennox Mac Ankrah, Kenneth K. Kye-Amoah, Mildred Adusei-Poku, Kwamena W. C. Sagoe, Jason T. Blackard

**Affiliations:** 1 Division of Gastroenterology & Hepatology, Department of Internal Medicine, University of Cincinnati College of Medicine 2514, Cincinnati, Ohio, USA; 2 Department of Medical Microbiology, Medical School, College of Health Sciences, University of Ghana 58835, Accra, Ghana; 3 Department of Microbiology and Immunology, School of Medicine, University of Health and Allied Sciences 549574, Ho, Ghana; 4 The Laboratory Unit, Sefwi Wiawso Municipal Hospital 59892, Sefwi Wiawso, Western North Region, Ghana; DOE Joint Genome Institute, Berkeley, California, USA

**Keywords:** human immunodeficiency virus, genetic diversity, genetic recombination

## Abstract

Here, we report the near-full-length genome sequence of HIV-1 strain BF14—a G/K/J unique recombinant form (URF)—obtained from the peripheral blood of a person living with HIV in Western North Region, Ghana. This genome adds to the limited near-full-length HIV-1 data from Ghana and documents ongoing inter-subtype recombination.

## ANNOUNCEMENT

Human immunodeficiency virus type 1 (HIV-1), a member of the family *Retroviridae* and genus *Lentivirus*, exhibits extensive genetic diversity with multiple subtypes and circulating recombinant forms (CRFs) that co-circulate globally (1). In Ghana, multiple HIV-1 subtypes circulate, including CRF02_AG, subtypes G, J, and K, creating opportunities for inter-subtype recombination (2, 3). Unique recombinant forms (URFs) continue to emerge (2–4) and complicate molecular surveillance and vaccine development efforts.

Peripheral blood (6 mL in EDTA tube) was collected in June 2025 from a 34-year-old woman attending Sefwi Wiawso Municipal Hospital in Sefwi, Western North Region, Ghana, who had been on tenofovir/lamivudine/dolutegravir (TLD) antiretroviral therapy for 5 years with an undetectable viral load as part of HIV surveillance in an area with limited surveillance data. Peripheral blood mononuclear cells were isolated by Ficoll-Paque PLUS (Cytiva) density-gradient centrifugation, and proviral DNA was extracted with the QIAamp DNA Blood Mini Kit (Qiagen). Near-full-length amplification used the previously published first-round primers SK145 and OFM19 (5) with the UltraRun Long Range PCR Kit (Qiagen). The single ~8.3 kb amplicon (spanning approximately HXB2 positions 1,358–9,632, i.e., partial *gag* through *nef*) was gel-purified (QIAEX II, Qiagen) and submitted for sequencing.

Libraries were prepared from 100 ng of product with the NEBNext Ultra II FS DNA Library Prep Kit and sequenced (2 × 61 bp) on an Illumina NextSeq 2000 at the University of Cincinnati Genomics, Epigenomics, and Sequencing Core. The core provided 2,800,402 raw read pairs that were quality- and adapter-filtered (no issues flagged during quality control). Human reads were removed by mapping to GRCh38 with Bowtie 2 (6), and the 166,401 non-human read pairs were assembled *de novo* with SPAdes v4.2.0 (7). All tools were run with default parameters unless otherwise specified. The HIV-1 genome was recovered as a single near-full-length contig (8,302 bp; 41.2% guanine–cytosine [GC]; no ambiguous bases; 191× SPAdes k-mer coverage; 2,236× mean read depth over 99.95% of the genome on remapping) (8). Intact *pol*, *env*, and *nef* open reading frames were identified by comparison with HXB2. The 5′ portion of *gag* and both LTRs were not captured and were unresolved.

**Fig 1:**
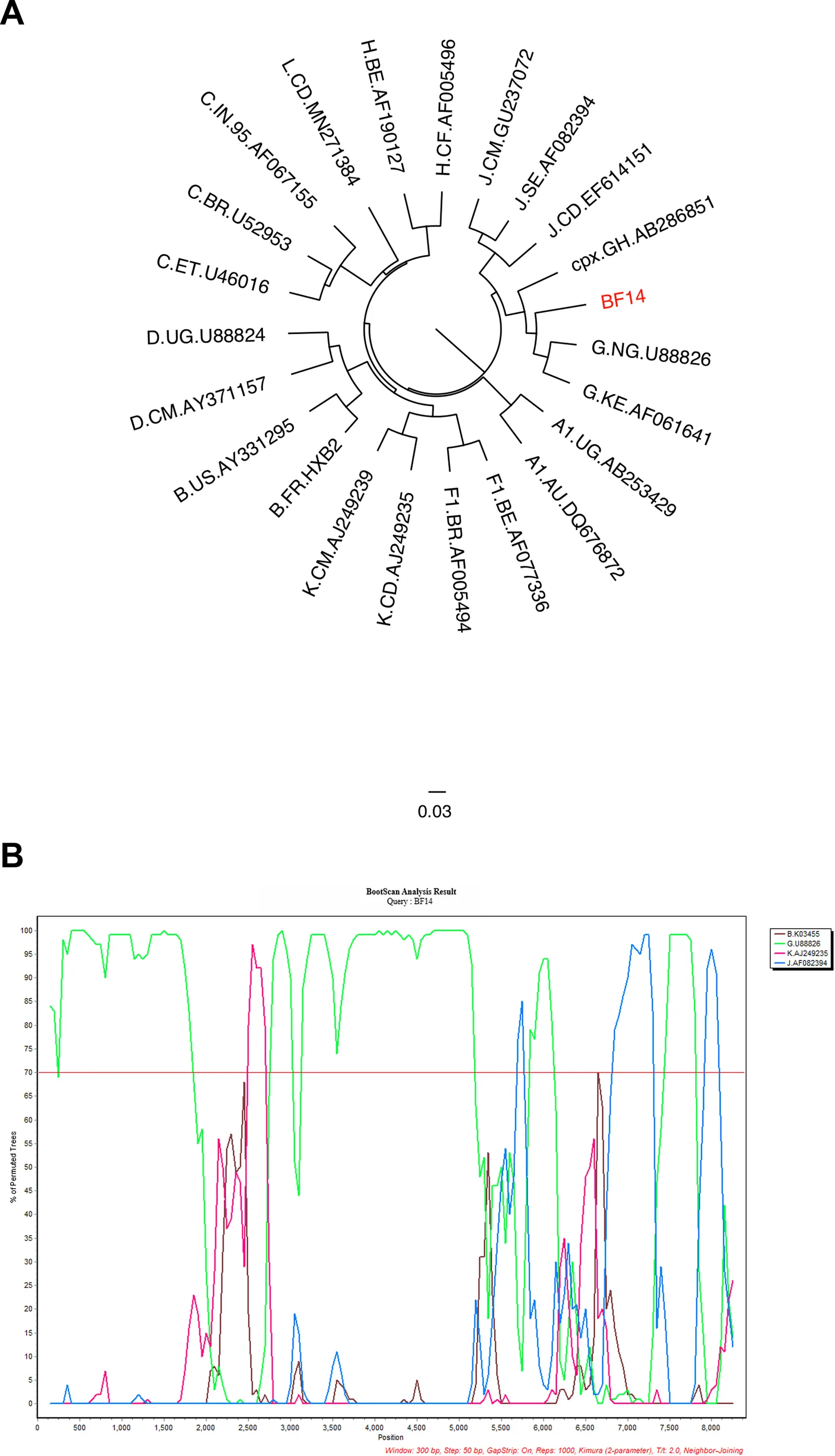
(**A**) Maximum-likelihood phylogenetic tree of the near-full-length genome of HIV-1 strain BF14 and reference sequences inferred in IQ-TREE 3 with 1,000 ultrafast bootstrap replicates. Strain BF14 groups with CRF06_cpx and is more distantly related to subtype G references. (**B**) BootScan analysis results (SimPlot v3.5.1; 300-bp window, 50-bp step) showing the G/K/J mosaic structure. The 70% permuted tree support is depicted by the dotted line and is the cut-off support to confirm recombination.

The near-full-length genome was aligned with 22 HIV-1 group M reference sequences using MAFFT v7.526 (9). The best-fit substitution model (GTR + F + R4) was selected with ModelFinder (10), and a maximum-likelihood tree was inferred in IQ-TREE 3 (v3.1.3) (11) with 1,000 ultrafast bootstrap replicates (12). The BF14 strain clustered with HIV-1 CRF06_cpx and more distantly with subtype G reference (Fig. 1A), showing highest nucleotide identity (90.0%) to CRF06_cpx strain from the Democratic Republic of the Congo (GenBank accession no. AB485661). However, recombination analysis using SimPlot (v3.5.1) (13) revealed a G/K/J mosaic structure (Fig. 1B). The predominant subtype G backbone likely accounts for phylogenetic clustering, while the K and J fragments represent minor recombinant regions. This URF highlights the ongoing diversification of HIV-1 in Ghana through inter-subtype recombination.

## Data Availability

The HIV-1 genome sequence reported here has been deposited in GenBank under accession number PZ490244. Raw sequencing reads have been deposited in the NCBI Sequence Read Archive (SRA) under BioProject accession number PRJNA1470555, with SRA accession number SRR40782179.
